# Fatty acid profiles and Delta9 desaturase (stearoyl-CoA desaturase; *SCD 1*) expression in adipose tissue surrounding benign and malignant breast tumors

**DOI:** 10.1016/j.heliyon.2023.e20658

**Published:** 2023-10-06

**Authors:** Reyhaneh Sefidabi, AliReza Alizadeh, Sadaf Alipour, Ramesh Omranipour, Maryam Shahhoseini, Amin Izadi, Samira Vesali, Ashraf Moini

**Affiliations:** a Breast Diseases Research Center (BDRC), Cancer Institute, Tehran University of Medical Sciences, Tehran, Iran; b Department of Embryology, Reproductive Biomedicine Research Center, Royan Institute for Reproductive Biomedicine, ACECR, Tehran, Iran; c Department of Surgery, Arash Women's Hospital, Tehran University of Medical Sciences, Tehran, Iran; d Department of Surgical Oncology, Cancer Institute, Tehran University of Medical Sciences, Tehran, Iran; e Department of Genetics, Reproductive Biomedicine Research Center, Royan Institute for Reproductive Biomedicine, ACECR, Tehran, Iran; f Department of Biochemistry, Faculty of Basic Sciences and Advanced Technologies in Biology, University of Science and Culture, Tehran, Iran; g Department of Cell and Molecular Biology, Faculty of Biology, College of Science, University of Tehran, Tehran, Iran; h Department of Basic and Population Based Studies in NCD, Reproductive Epidemiology Research Center, Royan Institute, ACECR, Tehran, Iran; i Department of Endocrinology and Female Infertility, Reproductive Biomedicine Research Center, Royan Institute for Reproductive Biomedicine, ACECR, Tehran, Iran; j Department of Gynecology and Obstetrics, Arash Women's Hospital, Tehran University of Medical Sciences, Tehran, Iran

**Keywords:** Adipose tissue, Benign breast disease, Breast neoplasms, Fatty acids, Stearoyl-CoA desaturase, Subcutaneous fat

## Abstract

The progression of tumors occurs through interactions between the tumor and the stroma. Understanding the role of adipose tissue (AT), as the main component of the breast tumor microenvironment (TME) in the development of cancer, is crucial for the early detection of breast cancer (BC). This study compared the FA profiles, desaturase index (DI), and stearoyl CoA desaturase 1 (*SCD1*) mRNA levels in the AT that surrounds tumors in women with BC and benign breast disease (BBD). Specimens were collected from 40 Iranian women who had undergone breast surgery. These women were age- and BMI-matched and were divided into two groups: BC (n = 20) and BBD (n = 20). Gas chromatography and quantitative real-time PCR were used to analyze the FA profiles and *SCD1* mRNA levels, respectively. The DI was calculated by dividing the amounts of monounsaturated FAs by the amount of saturated FA. There were no significant differences in age and BMI between women with BC and BBD. The FA profiles and DI were also similar in both groups. However, mRNA levels of *SCD1* were found to be 5 times higher in the breast AT of BC than in the breast AT of BBD (p < 0.0001). We showed that *SCD1* was significantly upregulated in the AT surrounding BC tumors, even though the DI and FA profiles were unchanged compared to those in the AT of BBD patients. It is important to note that the breast AT of women with BBD has previously been overlooked and warrants further studies.

## 1 Introduction

Breast cancer (BC) is the most commonly diagnosed cancer in women, with over two million new cases and approximately 680,000 related deaths worldwide in 2020 [1]. While the incidence is higher in Western societies, epidemiologic and clinical outcome data suggest that the rate of BC in developing countries is also rising and is associated with increased mortality [2]. In Iran, the age at diagnosis of BC patients is lower than in other comparable countries and much lower than in western countries [3]; this highlights the importance of effective detection methods for the early stages of BC at younger ages.

In recent years, the assessment of the tumor microenvironment (TME) has been incorporated into new approaches for managing BC, in addition to the traditional grading and typing based on the assessment of tumor cells alone [4]. BC development is highly dependent on the heterotypic interaction between tumor cells and stromal cells of the TME [5], especially for adipose tissue (AT) in this fat-rich organ. Understanding the composition of breast AT and the factors that influence this major component of breast tissue stroma is important for understanding its role in the etiology, risk, and progression of BC [6].

The role of AT, and more especially of adipocytes in BC progression and metastasis, is an emerging and critical new area of research. Recent studies [7,8] have enhanced our understanding of the importance of histologically normal AT as a major cellular component of breast TME. In recent years, several aspects of the involvement of adipocytes, particularly altered fatty acid (FA) composition and expression of genes related to lipid metabolism, in all phases of BC progression have elicited the attention of researchers working in the field of BC [9,10].

While the crucial roles of FA profiles in AT function have been reported [11], there is limited information available on the FA profile and metabolism in the AT surrounding various types of breast tumors. The link between AT accumulation and cancer, along with the role of FAs, offers interesting opportunities for the discovery of biomarkers and the development of nutritional strategies to control cancer, potentially in combination with therapies [12].

The nature of stored FAs in breast fat, which are necessary for normal differentiation and morphogenesis of breast epithelial cells, has been investigated since the early 1990s [[13], [14], [15]]. Furthermore, previous studies in several countries [16,17] have examined FA composition in tumor cells and TME. Recently, differences in the phenotype and signaling pathway of BC adipose microenvironment between AT attached to the primary tumor *vs*. AT distant (more than 2 cm) from the tumor have been reported [7]. While little information exists on the effective roles of FA metabolism in the AT surrounding various types of breast tumors, the past decade of in-depth research on the overexpression of stearoyl-CoA desaturase (*SCD1)* in BC cells has provided strong evidence for the major role of *SCD1* in tumor progression [10,18].

Previous studies have confirmed that alterations in lipid metabolism are a central feature of cancer cells, often manifesting as the excessive synthesis of specific FAs [19]. In this regard, recent and current studies have been focusing on the desaturase index (DI; monounsaturated fatty acids (MUFA)/saturated fatty acids (SFA) ratio) and SCD1 activity in AT to better understand FA metabolism and the roles of MUFA in health and diseases [20]. Moreover, AT is characterised by a high quantity of MUFA itself [21,22] and the increase of desaturase metabolism is also reported as biomarker in the shift from normal to overweight and obesity [23,24]. The activity of SCD1 has been estimated by the DI which is calculated using the MUFA: SFA ratio [palmitoleate (PMA): palmitate (PA) (desaturase C16: DI16) and oleate (OA): stearate (SA) (desaturase C18: DI18)]. Indeed, the increased conversion of SFA to MUFA, associated with increased SCD1 activity that can be monitored using the DI, is a possible feature of tumors [25,26]. Upregulation of the gene of this enzyme accelerates cell proliferation [25] and migration and remarkably enhances the ability of tumor formation [27]. Therefore, SCD1 expression may be an important prognostic factor for predicting the behavior of malignant and benign tumors.

To the best of our knowledge, the question of whether altered levels of SCD1 expression in AT of breast TME affect tumor behavior, proliferation, and invasion has not been addressed. However, in cancer cells the connection between SCD1 expression and malignancy (patients' shorter life expectancy) has been assigned [28] and Igal clarified all the reported data on the FA levels and SCD1. The objective of this study was to detect and compare FA composition, assess the differences in *SCD1* expression besides DI, and investigate the association between these parameters in breast AT surrounding benign and malignant tumors.

## 2 Materials and methods

### 2.1 Study population

This case-control study was conducted on Iranian women who underwent breast surgery at two referral hospitals affiliated with the Tehran University of Medical Sciences in Tehran, Iran, between May and December 2021. The study received ethical permission from the Ethics Committee of Tehran University of Medical Sciences (IR.TUMS.IKHC.REC.1399.502). The participants were age- and body mass index (BMI)-matched.

Before beginning the study, all participants were informed about the nature and purpose of the study, and written informed consent was obtained from each patient who provided tissue samples. Demographic and clinical characteristics were obtained through the demographic questionnaire and medical record. The study methodologies conformed to the standards set by the Declaration of Helsinki.

The inclusion criteria for this study included being between the ages of 20 and 70, not having diabetes, not having a history of radiation, surgery, or cytotoxic chemotherapy, and not having a history of smoking, drinking, taking supplements, or taking medications that could affect glucose and lipid metabolism, particularly FA supplements. Lactating mothers and pregnant women were excluded from the study. Eligible cases to participate in the study were divided into two groups: the BC group, consisting of women with the diagnosis of estrogen-receptor-positive invasive BC, and the BBD group comprising women diagnosed with histologically-proven benign breast lesions.

### 2.2 AT biopsies and storage

AT specimens were obtained from the tumor-free region of the breast subcutaneous AT located approximately 30–50 mm from the tumor in all participants, as suggested by *Mentoor* et al. [7] and *Ouldamer* et al. [17]. The obtained fat tissue sample was immediately rinsed using isotonic saline solution, segmented, plunged into liquid nitrogen (snap frozen), and placed in labeled cryovial tubes. Subsequently, all cryovials were stored at liquid nitrogen (−196 °C) until analysis [29].

### 2.3 Fatty acid analysis

All samples were thawed at room temperature for 2 min, segmented, and immediately methylated which suggested by Ostermann et al. [30]. Fatty acid methyl esters (FAME) were prepared with boron trifluoride (BF3) according to ISO 12966-2. A gas chromatograph (Shimadzu GC-2010 PLUS, Shimadzu Corporation, Japan) with a flame ionization detector (FID) was used to separate FAME. Separation was performed using a 60 m × 0.25 mm, 0.2 μm column (Dikmacap 2330). Hydrogen was used as the carrier gas at a column flow rate of 1.20 mL/min. The temperature of the column was increased from 60 to 240 °C within 30 min. The injector and detector temperatures were 250 °C and 280 °C, respectively. FAs were identified by comparing similar peak retention times (Rt) using pure FA standards, and the results are expressed as percentages accordingly.

### 2.4 RNA extraction, cDNA synthesis, and qPCR procedures

We focused on breast AT surrounding benign and malignant breast tumors. Total RNA was purified from all tissue samples using TRIZOL (QIAGEN, Germany) according to the manufacturer's protocol. The integrity of the extracted RNA was verified using 1% agarose gel electrophoresis. The extracted RNA quality was evaluated by a nanodrop (Thermo Scientific, USA) in terms of the A260/280 ratio. Briefly, cDNA synthesis was carried out on RNA samples using the PrimeScript RT Reagent Kit (Smo Bio, Japan) according to the manufacturer's protocol. Subsequently, an analysis of PCR products by gel electrophoresis was done. Quantification of mRNA was performed by qRT-PCR on the Step-One RT-PCR system (Applied Biosystems, USA) with the primer indicated as follows. All reactions were run in duplicates. The primer was designed for one target gene using the NCBI primer Blast and the Perl primer Software (version 1.1.21). The primer sequences, product sizes, and GenBank accession number for the sequences reported in this paper are listed in Table 1. To verify the specificity of each primer, melting curve analyses were performed. Standard curves, which were obtained by logarithmic dilution series of total cDNA, were generated for target gene to evaluate primer efficiency. Eventually, the housekeeping gene GAPDH was adopted to identify normalization and messenger RNA expression levels of the target gene were analyzed by quantitative real-time PCR (2^−Δ*C*T^).

**Table 1:** Sequences of the primers used for the quantification of the target and housekeeping genes.

Gene	Accession number	Sequence of the primers	Product size (bp)
*SCD1*	NC_000010.11	TGACGCTGATCCCTTCTGC AATAGTCAAGAAGATCCGCAG	**147**
GAPDH	NC_000012.12	TGAGAAGTATGACAACAGCCTC TGATGGCATGGACTGTGGT	**134**

### 2.5 Statistical analysis

Statistical analysis was performed using SPSS 22.0 software. Results were presented as mean ± SD and frequency (percent) that were all normally distributed. Student's t-test and one-way ANOVA test were applied for comparisons between BC and BBD patients. Pearson correlation analysis was used for the relationship between *SCD1*, DI18, and tumor size. A p-value of 0.05 was considered significant.

## 3 Results

The forty patients, aged 20–67 years, were divided into two groups: the BC group, consisting of 20 women with estrogen-receptor-positive invasive BC [including 18 invasive ductal carcinoma (IDC) and 2 invasive lobular carcinoma (ILC)] and the BBD group comprising women diagnosed with histologically-proven benign breast lesions. The mean (SD) age of women was 41.03 ± 11.74 years, and the mean BMI (SD) was 26.86 ± 4.95 kg/m^2^. Participants in the two groups were matched by age (p = 0.087) and BMI (p = 0.674). The mean age at menarche and age at first pregnancy were not statistically different between BC and BBD patients. Sixty percent of BC patients and 79% of BBD participants were parous (p = 0.2). There was no statistically significant difference between the two groups regarding the history of breastfeeding among parous women (p = 0.57). The family histories of BC and the menopausal statuses were similar in the two groups. Details of the demographic and clinical characteristics of all the participants are provided in Table 2A.

**Table 2:** Patient and tumor characteristics.

A. Demographic and clinical characteristics of patients studied			
	BC (n = 20)	BBD (n = 20)	*P*-value
**Age (yr)**			
Mean (SD)	44.20 (9.30)	37.85 (13.23)	0.087
**BMI (kg/m2)**			
Mean (SD)	27.19 (4.82)	26.52 (5.18)	0.674
**Age at menarche (yr)**			
Mean (SD)	13.77 (1.36)	12.64 (2.06)	0.109
**Parity, n (%)**			0.2
Nulliparous	8 (40%)	4 (21.1)	
Parous (1+ live births)	12 (60)	15 (78.9)	
**Age at first pregnancy**^a^**(yr)**			
Mean (SD)	23.57 (6.44)	22.54 (6.19)	0.675
**History of breastfeeding, n (%)**			0.57
No	8 (40%)	14 (70%)	
Yes	12 (60%)	6 (30%)	
**Menopausal status**^b^**, n (%)**			
Pre-/perimenopausal	17 (85%)	17 (85%)	
Postmenopausal	3 (15%)	3 (15%)	–
**Family history of breast cancer, n (%)**			
No	15 (75%)	15 (75%)	
Yes	5 (25%)	5 (25%)	–
**Chronic medical drug use, n (%)**			
No	9 (47.4%)	7 (35%)	0.433
Yes	10 (52.6%)	13 (65%)	

**B.** Clinical characteristics of breast lesions (Histologic diagnosis and characteristics of breast lesions)	
**BC samples (n = 20)**	
**Tumor size (mm)**	
Mean (SD)	38.26 (18.77)
**Histologic type, n (%)**	
Invasive ductal carcinoma (IDC)	18 (90%)
Invasive lobular carcinoma (ILC)	2 (10%)
**Biological marker**	
ER-positive/PR-positive, n (%)	17 (85%)
ER-positive/PR-negative, n (%)	3 (15%)
HER2-positive, n (%)	3 (15%)
HER2-negative, n (%)	17 (85%)
Ki-67 (%), Mean (SD)	21.97 (15.48)
**Histologic grade, n (%)**	
I	2 (10%)
II	16 (80%)
III	2 (10%)
**BBD samples (n = 20)**	
**Tumor size (mm)**	
Mean (SD)	17.82 (12.92)
**Histologic type, n (%)**	
Fibrocystic change (FCC)	7 (35%)
Fibroadenoma	8 (40%)
Others	5 (25%)

*Abbreviations: BMI* body mass index, *SD* standard deviation BC: Breast cancer, BBD: benign breast disease.

a Among parous women only.

b Among postmenopausal women only.

Data on tumor size revealed that there was a statistically significant difference in mean tumor size between the two groups (BBD: 17.82 ± 12.92 and BC: 38.26 ± 18.77 mm, p = 0.003). As shown in Table 2B, most of the BC patients showed a histological type of invasive ductal carcinoma (90%) and grade II (80%) with an ER-positive/PR-positive marker (85%). Forty percent of the BBD women showed a histological type of fibroadenoma.

The comparison of breast AT profiles of FAs in BC and BBD women is shown in Table 3. It was found that only arachidic acid concentration was statistically different between the groups (p = 0.010). The mean concentration of C20:0 was higher in BBD (0.31 ± 0.08) than in BC (0.25 ± 0.06) patients. Two desaturase indexes (C16:1/C16:0 and C18:1/C18:0) had similar in both groups (Table 4).

**Table 3:** Breast adipose tissue composition of fatty acids in breast cancer and benign breast disease women.

Fatty Acids (% of total fatty acids)	BC (n = 20)	BBD (n = 20)	*P*-Value*
12:0 (lauric acid)	0.24 ± 0.10	0.26 ± 0.14	0.509
14:0 (myristic acid)	1.55 ± 0.31	1.52 ± 0.39	0.800
15:0 (pentadecylic acid)	0.11 ± 0.06	0.13 ± 0.07	0.229
16:0 (palmitic acid)	21.38 ± 1.96	21.22 ± 2.66	0.832
17:0 (margaric acid)	0.29 ± 0.08	0.29 ± 0.10	0.986
18:0 (stearic acid)	4.72 ± 1.13	5.02 ± 1.33	0.453
20:0 (arachidic acid)	0.25 ± 0.06	0.31 ± 0.08	0.010*
22:0 (behenic acid)	0.37 ± 0.13	0.36 ± 0.14	0.816
24:0	0.26 ± 0.10	0.21 ± 0.14	0.295
**Total SFAs**	29.16 ± 2.63	29.33 ± 3.27	0.856
15:1	0.07 ± 0.03	0.07 ± 0.03	0.631
16:1	2.55 ± 0.85	2.33 ± 0.90	0.433
17:1	0.26 ± 0.08	0.25 ± 0.07	0.651
18:1t	0.47 ± 0.19	0.54 ± 0.15	0.237
18:1c/18:1 n-9 cis (oleic acid)	41.13 ± 2.57	41.21 ± 2.81	0.929
20:1	0.06 ± 0.06	0.06 ± 0.03	0.966
24:1	0.07 ± 0.05	0.11 ± 0.11	0.215
**Total MUFAs**	44.06 ± 2.90	43.90 ± 2.90	0.864
18:2 n-6 trans (Linolelaidic acid)	0.22 ± 0.1	0.25 ± 0.06	0.317
18:2 n-6 cis (linoleic acid)	21.89 ± 3.23	21.64 ± 2.78	0.797
18:2 n-6 c9 t11: CLA (rumenic acid)	0.80 ± 0.12	0.89 ± 0.22	0.093
20:2 n-6 (eicosadienoic acid)	0.43 ± 0.15	0.47 ± 0.10	0.287
20:4 n-6 (arachidonic acid)	0.50 ± 0.13	0.45 ± 0.23	0.436
**Total n-6 PUFAs**	22.36 ± 3.22	22.10 ± 2.78	0.778
18:3 n-3 (a-linolenic acid)	0.70 ± 0.17	0.73 ± 0.12	0.512
20:5 n-3 (eicosapentaenoic acid) EPA	0.07 ± 0.05	0.07 ± 0.11	0.985
22:5 n-3	0.15 ± 0.06	0.14 ± 0.08	0.891
22:6 n-3 (docosahexaenoic acid) DHA	0.10 ± 0.04	0.09 ± 0.04	0.367
**Total n-3 PUFAs**	1.00 ± 0.24	1.03 ± 0.20	0.623
**Total PUFAs**	23.79 ± 3.34	23.60 ± 2.83	0.846
n-6/n-3 PUFA ratio	23.68 ± 7.02	21.90 ± 4.10	0.333
Odd chain fatty acids	0.72 ± 0.19	0.74 ± 0.23	0.737
Other fatty acids	1.42 ± 0.35	1.37 ± 0.27	0.664
**Total fatty acids**	98.58 ± 0.36	98.62 ± 0.27	0.665

Values are presented as the mean ± SD. * obtained by independent *t*-test. Statistically significant level was 0.05.

BC: Breast cancer, BBD: benign breast disease.

**Table 4:** Desaturase index in breast adipose tissue of breast cancer and benign breast disease women (Mean ± SD).

	BC (n = 20)	BBD (n = 20)	*P*-Value*
C16:1/C16:0	0.19 ± 0.04	0.11 ± 0.05	0.640
C18:1/C18:0	9.26 ± 2.71	8.85 ± 2.73	0.639

BC: Breast cancer, BBD: benign breast disease * obtained by independent *t*-test. Statistically significant level was 0.05.

**Fig. 1:**
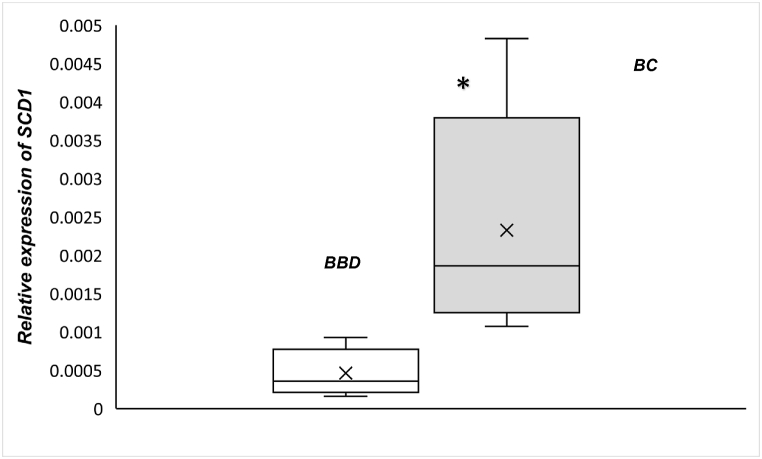
Box plot that shows the mRNA expression of *SCD1* in breast AT surrounding tumor of patients with breast cancer (BC; n = 20) and subjects with benign breast disease (BBD; n = 20). *P-value <0.0001.

As shown in Fig. 1, the mRNA expression of *SCD1* was significantly different between patients with BC and those with BDD (p < 0.0001). The mean relative expression level of *SCD1* in the BC group was 0.002358, while it was 0.000448 in the BBD group. Considering the difference in tumor size between the two groups, the correlation between *SCD1*, DI18 and tumor size quantitatively was assessed. The results of the correlation between *SCD1* gene expression, DI18, and tumor size in breast subcutaneous AT showed that among all participants there was only a statistically significant and positive relationship between DI18 and tumor size (r = 0.369, p = 0.045). In each group, there was no statistically significant correlation between *SCD1* expression, and tumor size, and between SCD1 expression and DI18 (Table 5A-C).

**Table 5:** Relationship between the mRNA expression levels of SCD1, DI18, and tumor size in breast AT surrounding tumor.

(A; BC+BBD; n = 40)				
		SCD1	DI18	Tumor size (mm)
SCD1	*r*		−0.141	+0.255
	*p*		0.385	0.174
DI18	*r*	−0.141		+ 0.369*
	*p*	0.385		0.045

**(B**; BC; n = 20**)**			
		SCD1	DES18
Tumor size (mm)	*r*	−0.194	+0.421
	*p*	0.427	0.073

**(C; BBD;** n = 20**)**			
		SCD1	DES18
Tumor size (mm)	*r*	0.382	+0.318
	*p*	0.246	0.341

Statistical significance using the Pearson correlation coefficient. *Significance at P < 0.05.

**Fig. 2:**
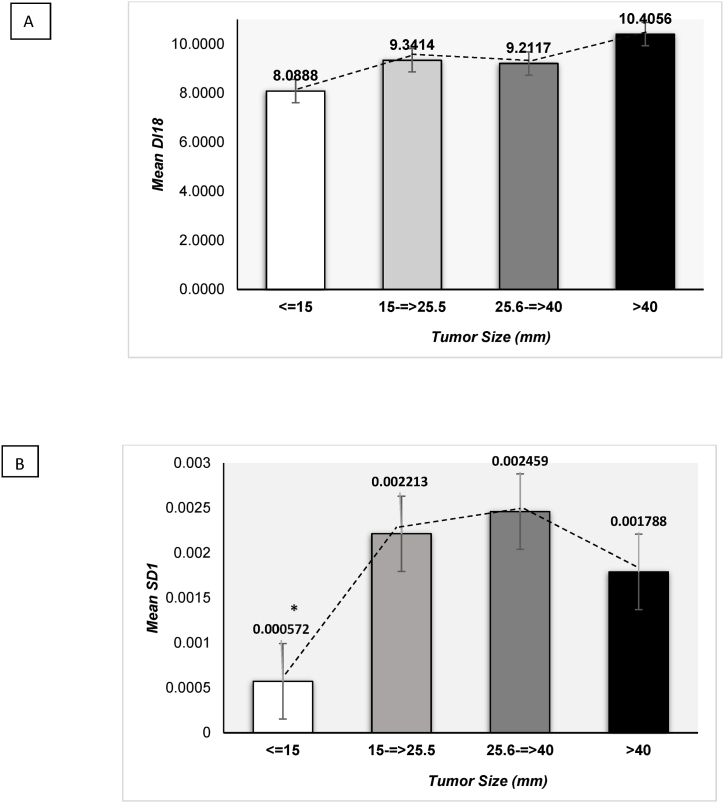
Mean comparison of desaturase index (DI18) (A) and *SCD1* (B) mRNA levels among all women (n = 40) studies by tumor size categories. *p < 0.05.

When tumor size was divided into quartiles (less than 15 mm, 15-equal/larger than 25.5 mm, 25.5 - equal/larger than 40 mm, and larger than 40 mm), only an increasing trend in mean DI18 by tumor size categories was observed among all women studied (p = 0.335) (Fig. 2a). However, among all the participants there was a statistically significant mean difference of *SCD1* by tumor size categories (p = 0.032). According to a Tukey posthoc analysis, the mean expression level of *SCD1* was higher in the category of 15–25.5 mm than the category <15 mm (p = 0.041) (Fig. 2b).

In women with BDD, there was not any significant mean difference of *SCD1*and DI18 by tumor size categories. In women with BC, similar to those with BDD, there was not any significant mean difference of *SCD1*and DI18 by tumor size categories.

## 4 Discussion

Our results provide considerable evidence regarding higher mRNA levels of *SCD1* in breast AT in BC, and a positive correlation between DI18 and tumor size among all participants. However, FA profiles as well as the desaturase indices (C16:1/C16:0 and C18:1/C18:0) were similar in both groups. There are some limitations to our research that should be considered when interpreting our findings, including the small sample size of this study due to the restrictions on eligible participants, who were matched for age and BMI in the BC and BBD groups. Nevertheless, the strength of the observed differences between the mRNA level of *SCD1* in breast AT of BC and BBD suggests that the sample size probably did not have a major impact on this factor in our study. In addition, the n-3 fatty acids concentration may be affected by acidic conditions and FA analysis method in current study**.**

FA profiles of AT were similar among experimental groups. The similar FA profiles may suggest that the long-time FA metabolism of subjects in both groups may have been similar in recent years. Additionally, we expected the omega-3 FAs in BC samples to be lower than in BBD samples. While malignant breast tumor behaviors were negatively associated with long-chain N-3 FAs in breast AT, our data do not support this hypothesis in TME which warrants further studies.

The differences in FA profiles of breast AT of cancer subjects are controversial. The somewhat contradictory results across studies investigating lipid profile and relative risk of BC may be due to the different genetic backgrounds of the participants and the differences in dietary intake habits in various populations and geographical areas that influence the FA composition of subcutaneous AT [31]. Several previous studies have examined the compositions of FAs in breast AT as a reflection of the type of dietary fat intake in recent years in different groups of normal, benign, and malignant women [32]. In addition, the low content of n-3 fatty acids in current study may be affected by acidic conditions**.** However, little is known about the significance of the alterations in FA composition between breast tumors and their adjacent ATs.

Studies investigating AT FAs profiles in BC have yielded some noteworthy findings in several countries all over the world [16,33]. There are limited data available on FAs profile in breast AT of women in Iran. There has only been a single case-control study that examined the FA composition of tumor samples versus adjacent normal-appearing tissue in this population [34]. To the best of our knowledge, no studies have compared the FAs profile of breast AT surrounding tumors between Iranian women with benign and malignant breast tumors.

The profile of major FAs in breast AT in our study was within the range expected on the basis of previous studies in Brazil [16] and France [17]. In line with our results, two case-control studies conducted in North America by *London* et al. in the Boston area [13]*,* and *Petrek* et al. in New York [14] in a population of American women revealed no association between the composition of specific FAs, particularly polyunsaturated and trans FAs, and risk for malignant or BBD. In contrast, our results differ from some previous European studies conducted in Finland [15], and the EURAMIC study in five European countries [35], which found differences in FA profiles in AT of BC subjects.

One of the possible reasons for the above results is the differences in analytical methods used for FA analysis. Due to the relatively low basal levels of some FA in AT surrounding breast tumors, and given the lack of homogeneity in sample preparation and analytical methods, FA profile assessment techniques such as GC or other routine methods may not be sensitive enough to detect minor differences. There is a need to develop more sensitive and uniform experimental methods for sample collection and preparation, and analysis of FA profiles in order to better understand the differences in FAs in AT surrounding various types of tumors in different countries. In addition, available literature suggested evidence of a difference between FA composition obtained from the tumor, its interface tissue, and grossly normal breast tissue in female BC patients [36]. Thus, alteration observed in the FA concentration of breast AT in different studies may be due to the different sampling locations.

Some studies have also looked at the specific FA profile of breast AT in the TME, taking into account the menopausal status [37]. In this regard, the current study observed the same distribution between the two groups by menopausal status and found no significant association between the FA composition of breast AT and the risk of BC among all women. These findings support most previous studies that did not identify a difference in total polyunsaturated fatty acids (PUFA) between patients with BC and healthy women, regardless of their menopausal status [13,37].

The findings of De Bree and colleagues [33] confirm the role of long-chain n-3 PUFAs intake in promoting the development of benign biological behavior and not contributing to the induction of breast tumors in pre-menopausal women. However, in contrast to our findings, they found that premenopausal patients with BBD had higher total PUFA levels than those with BC. Further studies that specifically consider premenopausal and menopausal status may help to address this discrepancy. Discriminate PUFA omega-6 and omega-3 for their significance in the tumor development and invasiveness, that is instead very well-known also in breast cancer. While we focused on AT around tumors, there is an interesting report on the analysis of the FA profiles in breast cancer patients comparing distal AT and proximal tumoral tissues taken from the same patient, which eliminates the effect of the diet [38]. Considering that the tumoral tissue contains the direct influence of the disease, such comparison was useful to shown the depletion of the omega-3 in the proximal tissue not depending from the diet. This is an important information to convince that not only the appropriate chemical reagents must be chosen for performing fatty acid profiles but also omega-3 are important elements that discriminate also the prognosis of cancer therapies, as reported [39]. Altogether, the FA profiles of AT were similar among our experimental groups. Future studies that carefully control technical procedures and consider sampling location and menopausal status may help to determine any differences in FA profiles between AT surrounding breast tumors.

One surprising finding from our study was the similarity in the Desaturase Index (DI) among the experimental groups. We had initially expected that BC samples, due to the overexpression of SCD1, would have a higher DI, as has been observed in other studies of abnormal conditions. SCD1 in mammals affects a variety of key physiological variables. SCD1 is the predominant enzyme isoform in human lipogenic tissues and is a delta-9 desaturase enzyme, responsible for the conversion of SFAs to MUFAs [20]. A previous study found that Brazilian women with BC had a higher DI18 than the BBD; this was attributed to the novel functions of the enzyme *SCD1* that is overexpressed in Brazilian women with BC [16]. Although the DIs are similar, the present study provides noticeable differences between the expression of SCD1 in AT samples of BC patients compared to BBD women. Despite the increased expression of *SCD1* in the breast AT of BC patients compared to BBD (control) patients, DI remained unaltered.

Recently, in light of the progress in understanding the influences of TME on tumor development, the contribution of TME to tumorigenesis is ascribed to the differential gene expression patterns that support tumor growth and dissemination in invasive breasts compared to non-malignant breasts [10]. The past decade of in-depth research on the overexpression of *SCD1* in BC cells has provided strong evidence for the major role of *SCD1* in tumor progression [10,18,26].

Despite the recognition of the importance of *SCD1*, its role in the transformation of a normal cell and healthy mammary AT to a malignant state is unknown. To our knowledge, no study has compared the levels of *SCD1* expression specifically in the AT surrounding BC cells, and relative differences in the breast AT surrounding non-malignant tumors.

Interestingly, BC women in the current study showed a 5-fold higher mRNA level of *SCD1* in AT surrounding tumors compared to those with BBD. The current data confirm the previous findings [28,40] that reported the overexpression of several FA metabolism-related genes and reprogramming of lipid metabolism in the breast epithelial AT susceptible to cancer development. It should be noted that adjacent and distant AT co-exist within a malignant breast and may be subjected to similar local influences from the tumor. However, the differences in gene expression between the two tissues reflect the distance from the tumor [10].

Similar to our findings, previous research revealed the altered expression of *SCD1* in BC cells [28]. It has been pointed out that the overexpression of the genes leads to a major reprogramming of cellular energetics which accelerates a survival signal that enables tumor recurrence and BC progression [40]. This hypothesis is supported by prior research in which the expression levels of SCD1 were compared in BC cells and adjacent normal tissue after knockdown or chemical inhibition of SCD1. These studies demonstrated the central role of SCD1 in the development of breast tumors [34].

Previous studies suggest that for tumor metastasis and recurrence, a cancer cell must evade adjuvant therapies and survive in a new microenvironment [41]. Given the direct contact between cancer cells and adipocytes and evidence supporting the ability of adipocytes to sequester cancer drugs, it is important to focus on the upregulation of *SCD1* in AT of TME in BC. This issue could also lead to chemotherapy depotentiation besides gene expression changes in AT that may affect clinical outcomes of invasive BC. On the other hand, previous studies [26] have suggested defining the role of *SCD1* in the malignant processes of BC and evaluating the potential for *SCD1* as a therapeutic target. In this regard, *Holder* et al. suggested that *SCD1* expression varies by BC subtype. Furthermore, they reported that the levels of *SCD1* expression are associated with significantly shorter relapse-free survival (RFS) and overall survival (OS) in BC [28]. Higher mRNA levels of this enzyme reflect underlying biological alterations and thus constitute a possibly promising target for new therapeutic strategies.

The findings of this research work further support the idea of exposure of tumor cells to numerous altered forces and signals from the surrounding microenvironment leading to modification of their behavior and therefore tumor development [42]. Thus, the results show that the increased *de novo* FA synthesis is important for tumorigenesis [43] and also suggest that the function of *SCD1* may be modulated in the AT of TME of malignant cells compared to AT surrounding benign cells. The idea that the levels of different *SCD1* in different ATs surrounding tumors could be biomarkers for BC is an exciting idea for future research and clinical studies. However, AT is not the most available tissue to withdraw from cancer patients, whereas the data on red blood cell membranes are equally useful to estimate desaturase enzyme activities and are indeed the best candidate for the follow-up of cancer patients [12,21,23].

Limited studies have been conducted on the association between FAs and the risk of developing a specific subtype of estrogen receptor (ER) and progesterone receptor (PR) BC. Epidemiologic studies have suggested a strong correlation between elevated risk of ER+ BC cells and several lifestyle risk factors, such as fat intake, higher BMI, earlier age at menarche, and nulliparity [44]. With regards to ER+ cases in the current study, our results confirmed earlier research, which showed that Estradiol induces *SCD1* expression in ER+ BC [45]. It seems that increase in *SCD1* expression in the TME of ER+ breast carcinoma cells in current study is contradict with previous research suggests that estrogen acts as a repressor of *SCD1* in AT [46]. As mentioned earlier, there was no difference in the DI and FA profile among BC patients, regarding of their estrogen and progesterone receptor status. However, due to the small sample size, we were unable to analyze the heterogeneity between ER and PR subgroups, which warrants further studies on the subject. Overall, our findings support previous research on the important role of *SCD1* in estrogen-induced cell proliferation and suggest that *SCD1* may be a potential therapeutic target for estrogen-sensitive BC.

It is interesting to find a positive correlation between tumor size (less than 15 mm, 15-equal/larger than 25.5 mm, 25.6 – equal/larger than 40 mm, larger than 40 mm) and DI18. Among all participants the mean DI18 levels increased as the tumor size increased. Furthermore, a statistically significant decrease in mean *SCD1* levels was observed in tumors less than 15 mm compared to those between 15 and 25.5 mm. This remarkable decrease in mean *SCD1* and slightly lower DI18 in breast AT surrounding tumor 15 mm in size, may suggest that the signaling within the TME plays a role in breast tumor formation and that DI18 and *SCD1* may contribute to accelerated cell proliferation that support the hypothesis [47] targeting SCD1 may provide a novel therapeutic strategy for cancer treatment.

## 5 Conclusion

In conclusion, our study suggests a potential role of breast AT in TME, a major regulator of carcinogenesis in this fat-rich organ. This is the first report to show that *SCD1* is significantly upregulated in breast AT surrounding malignant tumors compared to benign tumors, as well as pointed out FA profiles. Our study paves the way for further research on how breast AT may promote the malignant progression of BC cells through the elevated expression of *SCD1*. It is important to note that further studies on the functional activity of the SCD enzyme as well as lipidomics profiles are needed.

## Funding

This project was financially supported by the Breast Diseases Research Center (BDRC), 10.13039/501100016287Cancer Institute, 10.13039/501100004484Tehran University of Medical Sciences, Tehran, Iran.

## Ethics approval and consent to participate

After obtaining permission from the Ethics Committee of Tehran University of Medical Sciences, Tehran, Iran (IR.TUMS.IKHC.REC.1399.502), samples and demographic data were collected from two hospitals in Tehran, Iran. Signed informed consent was obtained from all subjects.

## Consent for publication

This paper did not contain any individual person's data in any form (including any individual details, images, or videos).

## Data availability statement

All data used and analyzed during the current study are available from the corresponding author upon reasonable requests.

## CRediT authorship contribution statement

**Reyhaneh Sefidabi:** Data curation, Investigation, Methodology, Validation, Writing – original draft, Writing – review & editing. **AliReza Alizadeh:** Conceptualization, Investigation, Methodology, Validation, Writing – original draft, Writing – review & editing, Supervision. **Sadaf Alipour:** Data curation, Methodology, Validation, Writing – review & editing. **Ramesh Omranipour:** Data curation, Methodology, Validation, Writing – review & editing. **Maryam Shahhoseini:** Formal analysis, Investigation, Validation, Writing – review & editing. **Amin Izadi:** Methodology, Software, Writing – original draft. **Samira Vesali:** Formal analysis, Software, Validation, Writing – original draft. **Ashraf Moini:** Conceptualization, Funding acquisition, Project administration, Supervision, Writing – review & editing.

## Declaration of competing interest

The authors declare that they have no known competing financial interests or personal relationships that could have appeared to influence the work reported in this paper.

## Abbreviations

AT: adipose tissue
BC: breast cancer
BBD: benign breast disease
FA: fatty acid
SFAs: saturated fatty acids
MUFAs: monounsaturated fatty acids
PUFA: polyunsaturated fatty acids
IDC: invasive ductal carcinoma
ILC: invasive lobular carcinoma
TME: tumor micromovement
SCD1: stearoyl-CoA desaturase
ER: estrogen-receptor
PR: progesterone-receptor
PA: palmitate
SA: stearate
PMA: palmitoleate
OA: oleate
DI: desaturase index
DI16: desaturase C16
DI18: desaturase C18
ACC: acetyl-CoA carboxylase
FAS: fatty acid synthase
BF3: boron trifluoride
FID: flame ionization detector
Rt: retention times
RFS: relapse-free survival
OS: overall survival
FAME: fatty acid methyl esters
